# Regional hippocampal thinning and gyrification abnormalities and associated cognition in children with prenatal alcohol exposure

**DOI:** 10.1186/s11689-025-09595-8

**Published:** 2025-02-05

**Authors:** Blake A. Gimbel, Jeffrey R. Wozniak, Bryon A. Mueller, Kent A. Tuominen, Abigail M. Ernst, Mary E. Anthony, Erik de Water, the CIFASD, Donovan J. Roediger

**Affiliations:** 1 Nationwide Children’s Hospital, Columbus, USA; 2 The Ohio State University, Columbus, USA; 3 University of Minnesota Twin Cities, Minneapolis, USA; 4 Great Lakes Neurobehavioral Center, Edina, USA

**Keywords:** Fetal alcohol spectrum disorders, Prenatal alcohol exposure, Cognition, Memory, Hippocampal thickness, Hippocampal gyrification

## Abstract

**Background:**

Prenatal alcohol exposure (PAE) impacts hippocampal structure and function, contributing to deficits in memory and decision-making in affected individuals. Here, we evaluate hippocampal anomalies in children with PAE and an unexposed comparison group using advanced MRI methods that characterize hippocampal curvature and thickness.

**Methods:**

Participants, ages 8 to 16 years, included children with PAE (*n* = 48) and an unexposed comparison group (*n* = 46) who underwent a dysmorphology exam, neuropsychological assessment, and an MRI scan. Height, weight, head circumference, and dysmorphic facial features were evaluated. Of those with PAE, 4.2% had fetal alcohol syndrome (FAS), 22.9% had partial FAS, and 72.9% had alcohol-related neurodevelopmental disorder. Neuropsychological testing included measures of intelligence and memory functioning. T1-weighted anatomical data were processed with the Hippunfold pipeline, which “unfolds” the complex hippocampal structure onto a template surface and provides measures of thickness and gyrification/curvature at each vertex. Permutation Analysis of Linear Models (PALM) was used to test for group differences (PAE vs. comparison) in hippocampal thickness and gyrification at each vertex and also to assess correlations with cognitive functioning.

**Results:**

There were significant regional differences in thickness and gyrification across bilateral hippocampi, with the PAE group showing substantially thinner tissue and less curvature than the comparison group, especially in CA1 and subiculum regions. For those with PAE, thinner subicular tissue (bilateral) was associated with lower IQ. Also in the PAE group, lower episodic memory performance was associated with thinness in the right hippocampus, especially in the subiculum region. There were no significant regional hippocampal patterns that were associated with cognitive functioning for individuals in the unexposed comparison group.

**Conclusions:**

We used a novel MRI method to evaluate hippocampal structure in children with PAE and an unexposed comparison group. The data suggest that PAE disrupts hippocampal development, impacting both the early-stage folding of the structure and its ultimate thickness. The data also demonstrate that these developmental anomalies have functional consequences in terms of core memory functions as well as global intellectual functioning in children with PAE.

**Supplementary Information:**

The online version contains supplementary material available at 10.1186/s11689-025-09595-8.

## Background

Prenatal alcohol exposure (PAE) is teratogenic, has wide-spread effects on brain development, and can result in fetal alcohol spectrum disorders (FASD)—lifelong neurodevelopmental conditions associated with cognitive and behavioral impairment [1]. Individuals with PAE have low total brain volumes, regional structural anomalies, abnormalities in cortical thickness and shape, white matter microstructural abnormalities, and altered neurodevelopmental trajectories across the lifespan [2, 3]. FASDs are common conditions with an estimated worldwide prevalence of 0.8% in the general population [4, 5] and 2.0 to 5.0% of the European and North American populations [5, 6]. They affect 13.6 to 28% of high risk rural populations in South Africa [7, 8]. Neurocognitive deficits are a core feature of FASD, ranging from broad intellectual impairment to select deficits in attention, executive functioning, memory, visual-perceptual/motor skills, and academic skills [9]. Relevant to the current study, a range of memory deficits are common in individuals with FASD, even when overall intellectual functioning is intact [10–12]. Memory impairments are particularly insidious because of their cascading impact on all aspects of learning and maturation over the course of neurodevelopment including eventual academic, social, and self-care skills. Neuroimaging methods such as magnetic resonance imaging (MRI) have repeatedly identified structural anomalies in subcortical regions such as the caudate and hippocampus in individuals with PAE [13–15].

The hippocampus is a critical subcortical structure involved in diverse cognitive functions including learning and memory, emotional processing, and aspects of executive functioning. Its development begins during the first months of life, continues rapidly into the 2nd year of life, and then continues at a slower, progressive pace into the 3rd and 4th years of life [16, 17]. The human hippocampus initially appears as a rudimentary flat form around 10 weeks of gestation. Thickening of the dentate gyrus region induces a rotation of the cornu ammonis (CA), resulting in infolding of the structure and deepening of the hippocampal sulcus [18]. As thickening continues, the structure eventually folds onto itself, resulting in a C-shaped form with a distinct sulcus similar to a cortical sulcus. By approximately 18 to 21 weeks gestation, the hippocampus is typically “adult-like” in its basic structure [19] (for comprehensive review, see [20]).

The hippocampus is exquisitely sensitive to a variety of developmental insults (e.g., hypoxia, malnutrition, stress, etc.) and is implicated in a variety of neurodevelopmental conditions including autism spectrum disorder, fragile X syndrome, and Down syndrome [21]. Hippocampal damage resulting from PAE and associated learning and memory impairments have consistently been described in individuals with FASD [22, 23]. Findings have included reduced hippocampal volume as well as shape abnormalities [24–26]. In addition, a robust preclinical literature using animal models has documented hippocampal injury resulting from PAE including altered neurogenesis [27] and decreased cell counts and/or reduced dendrite density (e.g., in the CA1 region of the hippocampus) [28, 29]. As a key manifestation of alcohol teratogenesis, hippocampal anomalies are thought to play an important role in the well-documented impairments in learning, memory, and cognition that occur frequently in individuals with FASD [11, 22, 30]. Importantly, recent qualitative research in adults living with FASD identifies pervasive daily memory challenges as common [31], highlighting the long term consequences of early hippocampal developmental disruption. Given its “keystone” role in cognitive development, the hippocampus is hypothesized to be a valuable potential target for early neurodevelopmental interventions such as choline—an essential nutrient that facilitates early brain development and supports neuronal functioning, especially in the hippocampus [32]. Maternal gestational choline supplementation has been shown to mitigate the effects of PAE on recognition memory and subcortical volumes [33]. Our own studies have shown that postnatal choline supplementation in young children ages 2–5 years with PAE improves aspects of learning, memory, cognitive function, and behavior regulation [34–36].

In many studies of both unexposed typically-developing children and those with neurodevelopmental conditions, the hippocampus is often treated as a single subcortical structure (e.g., total hippocampal volume as an outcome measure). For studies that parse the hippocampus, it is often segmented along its longitudinal axis into rough, somewhat arbitrary divisions (e.g., head, body, and tail) [37]. However, detailed histology reveals that the hippocampus is actually composed of a complex folded archicortical ‘ribbon’ that is contiguous with the cerebral cortex. Based on cyto-, myelo-, and chemoarchitectural features, the hippocampus is comprised of distinct subfields including the dentate gyrus; CA1, CA2, CA3, and CA4; the subiculum and presubiculum; and the hippocampal tail [38, 39]. These subfields show distinctive patterns of connectivity with other brain structures [20] and undergo diverse, non-linear trajectories of development from childhood to adulthood [40]. This complex morphology is highly variable across individuals [41]. Previously, we showed regional variability in hippocampal volume anomalies across subfields in PAE [42]. In youth with PAE ages 8 to16 years, volumes in 5 out of 10 hippocampal subfields including CA1, CA4, subiculum, presubiculum, and the hippocampal tail, were significantly smaller compared to unexposed comparison children. A limitation of that study, however, was that it measured volumes of hippocampal subfields without taking into account complex hippocampal morphology, which includes gyrification and thickness.

Novel tools that characterize individual-specific hippocampal folding at the regional level [38, 41] provide an important opportunity for further quantifying structural anomalies associated with PAE. Such approaches that reflect key early developmental processes hold promise for providing insights into critical periods of altered hippocampal development in the context of PAE and for tracking potential treatment-related neuroanatomical changes following hippocampus-targeted interventions. Here, we build on previous hippocampal volumetric work by using a novel MRI tool, HippUnfold [41], which “unfolds” the complex hippocampal structure onto a template surface, in order to better characterize hippocampal morphology in youth with PAE compared to unexposed comparison children. This novel approach to modeling MRI images of the hippocampus provides crucial advantages in accounting for individual differences in hippocampal folding structure that are known to influence hippocampal subfields as defined by manual segmentation [38]. The unfolding technique afforded by the HippUnfold tool provides more detailed measures of hippocampal morphology, and we leverage a vertex-wise analytic approach that provides high density regional specificity. An additional goal of this study is to examine associations between regional hippocampal morphology and neurocognitive function, and the method’s high level of resolution is ideal for understanding the neurodevelopmental impact of PAE on a structure serving complex cognitive functions. We hypothesized that hippocampal tissue would be atypically thin and that the structure would be atypically flat in children with PAE vs. comparison participants and that these anomalies would be associated with related cognitive functioning, especially in the memory domain.

## Methods

### Participants

A total of 96 participants, ages 8 to16 years, were enrolled in the study as part of the Collaborative Initiative on Fetal Alcohol Spectrum Disorders (CIFASD) (see www.cifasd.org). We used data collected at the University of Minnesota during the first visit of the larger longitudinal study. Recruitment took place between 2017 and 2019. Participants and their parent or guardian completed assent and consent processes and received monetary compensation. All study procedures were approved by the University of Minnesota Institutional Review Board. Participants in the PAE group were recruited by referral from the University of Minnesota Fetal Alcohol Spectrum Disorders Clinic in addition to community postings, external clinics and self-referral. Participants in the unexposed comparison group were recruited with advertisements online and provided at local community events and mailings to participants from previous University of Minnesota studies.

Exclusion criteria for this study were the presence of drug or alcohol misuse by the participant, severe neurological or developmental disorders (i.e., autism spectrum disorder, cerebral palsy, epilepsy or another neurological disorder affecting cognitive functioning), extremely low birth weight (< 1500 g), or MRI contraindications. Comorbid prenatal drug exposures in the PAE group were not considered exclusionary because many participants who also have prenatal alcohol exposure have at least one drug exposure as well and excluding these participants would limit our sample size. Other exposures in our PAE sample include methamphetamine, cocaine, marijuana, opioids, and tobacco. However, for the unexposed comparison group, prenatal substance exposure other than caffeine and tobacco was exclusionary.

One participant in the PAE group was excluded from analyses due to artifacts impacting MRI acquisition quality and one participant in the comparison group refused the scan, resulting in a total cohort of 94 for this study (48 PAE, 46 comparison).

### FASD diagnostic classification

Prior to being enrolled in the study, a phone screen and record review were completed to determine the history of PAE. Eligibility criteria for the PAE group included documented evidence of heavy PAE (≥ 13 drinks/week or ≥ 4 successive drinks during ≥ 1 week during pregnancy). Individuals without documented PAE who met diagnostic criteria for fetal alcohol syndrome (FAS) or partial fetal alcohol syndrome (PFAS) based on dysmorphology, growth characteristics, and cognitive impairments were also included in the PAE group as these features are relatively specific indicators of PAE [43, 44]. Participants completed a physical assessment conducted by one of two trained investigators (KLJ and JRW) to obtain ratings of the upper lip and philtrum, and measurements of palpebral fissure length (PFL), height, weight, and occipitofrontal circumference (OFC). The Modified Institute of Medicine criteria for FASD [45] were used for diagnostic classification. Normative data from Nelhaus et al. [46] were used to identify OFC abnormalities, and CDC Growth Charts [47] were used to quantify growth deficiency (≤ 10th percentile in height or weight for age and sex). For the purposes of diagnostic classification, neurobehavioral impairment was defined by 1) global intellectual functioning ≤ 78, or 2) two or more domains of impairment on individual cognitive measures (standard scores ≥ 1.5 standard deviations [SD] below the mean) and/or parent-report behavioral measures. Parent-report measures were used to identify anomalies in behavior and adaptive functioning. At least one objective measure of cognitive impairment was required to meet the diagnostic criterion for neurobehavioral impairment.

### Neurobehavioral evaluation

**Fig. 1:**
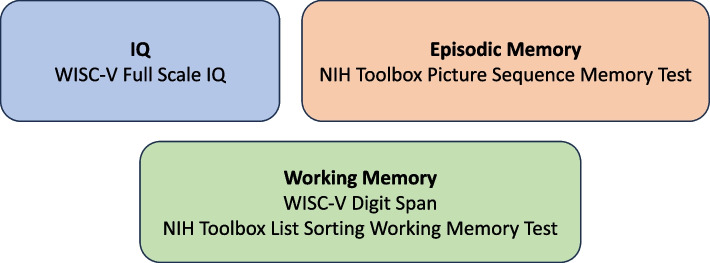
Domains of neurobehavioral assessment and selected measures. *Note:* WISC-V, Wechsler Intelligence Scale for Children, 5th Edition

All participants were administered a battery of assessments measuring general intelligence (full-scale IQ, hereafter IQ), working memory, and episodic memory (Fig. 1**)**. IQ was measured with the Wechsler Intelligence Scale for Children, 5th Edition ^24^ (WISC-V), which yields standardized scores (M = 100, SD = 15). The Wechsler Digit Span subtest (yielding scaled scores, M = 10, SD = 3) was used to measure working memory. Lastly, The NIH Toolbox [48] List Sorting Working Memory Test (LST) and Picture Sequence Memory Test (PSMT), which requires associative learning and recall of both visual and verbal information, were used to measure working memory and episodic memory, respectively (these tests yield T-scores, M = 50, SD = 10).

### MRI acquisition and processing

Structural MRI data were acquired on a Siemens 3 T Prisma scanner (Siemens, Erlangen, Germany) at the University of Minnesota’s Center for Magnetic Resonance Research equipped with a standard 32-channel head coil. T1-weighted and T2-weighted scans were acquired using custom pulse sequences chosen to match those used in the Lifespan Human Connectome Project Development (HCP-D) project [49] including automatic real-time motion detection and k-space line rejection and replacement software. T1-weighted scans were acquired as follows: multiecho MP-RAGE sequence with TR = 2500 ms, TE = 1.8/3.6/ 5.4/7.2 ms, TI = 1000 ms, voxel size = 0.8 mm isotropic, and flip angle = 8 degrees. Data were visually inspected to ensure accuracy by a trained operator (DJR). To assess for group differences in within-scanner motion in the remaining participants, we used the Euler value averaged across hemispheres [50]. The hippocampus was reconstructed in surface space from the T1-weighted images using Hippunfold [41], a containerized pipeline. Data from T2-weighted scans are not used by HippUnfold and were therefore not included in the current analyses. The Hippunfold pipeline includes intensity correction and template registration of the T1w image, hippocampal segmentation via a UNet neural network model, reconstruction of the hippocampi as 3D meshes in GIFTI format, and the calculation of dense scalar maps (containing 7,262 vertices for each of the two hippocampi) for morphometric measurements including thickness and gyrification. Here, gyrification is calculated from the ratio of surface area in MRI native space to surface area in the “unfolded” space [51]. Therefore, gyrification represents the degree of curvature/folding present in the hippocampal structure prior to unfolding.

### Statistical analysis

Statistical analyses were carried out using R version 4.1.1 [52] and Permutation Analysis of Linear Models (PALM) [53], a flexible, non-parametric statistical package that allows for permutation testing of dense (vertex-wise) brain measurements. Demographic characteristics of the sample and neurocognitive performance were tested for group differences (PAE vs. unexposed comparison) with chi-square tests and independent samples *t*-tests. Independent samples *t-*tests were used to examine group differences in within-scanner motion (Euler values). The advantages of PALM over a traditional parametric statistical approach include exact control of false positives (control for multiple comparisons) as well as statistical robustness regardless of data normality. Additionally, PALM supports threshold-free cluster enhancement (TFCE), which we utilized for all analyses. TFCE is a method for enhancing cluster-like structures in statistical maps without the need for arbitrary, predefined thresholds. A two-sample unpaired *t-*test was performed in PALM, at each vertex, to compare hippocampal thickness maps in PAE vs comparisons. PALM was also used to generate Pearson correlation maps showing, at each vertex, the extent to which hippocampal thickness correlated with neurocognitive scores (IQ, digit span, LST, and PSMT). Age and IQ were tested as potential confounding variables with two steps: 1) testing for group differences, and 2) testing for association with the outcome variable of interest.

## Results

The PAE and unexposed comparison groups were well-matched on age, sex, ethnicity, and handedness (Table 1). Because there was no significant group difference in age, it was not considered a potential confounding variable for subsequent analyses. In terms of race, the PAE group differed significantly from the unexposed comparison group: the PAE group had more participants who identified as Black or African American and Multiracial while the unexposed comparison group primarily consisted of participants identified as White. As expected, the participants in the PAE group had significantly more growth deficiency, microcephaly, and dysmorphic facial features than unexposed comparison participants.

**Table 1:** Demographic characteristics of participants included in the analyses

	PAE (*n* = 48)	Non-exposed comparison group (*n* = 46)	Statistical Test
*Age [M(SD)]*	12.4 (2.4)	12.7 (2.6)	*t*(92) = −0.600, *p* = 0.550
*Sex [n(%Female)]*	26 (54.2%)	22 (47.8%)	χ^2^ = 0.17, *p* = 0.683
*Ethnicity [n(%Hispanic)]*	2 (4.2%)	3 (6.5%)	χ^2^ < 0.01, *p* = 0.961
*Race*			
* [n(%American Indian/* *Alaska Native)]*	3 (6.2%)	0 (0%)	χ^2^ = 1.29, *p* = 0.256
* [n(%Asian)]*	2 (4.2%)	1 (2.2%)	χ^2^ = 0.00, *p* = 1.0
* [n(%Black or African* *American)]*	7 (14.6%)	0 (0%)	χ^2^ = 5.29, *p* = 0.022
* [n(%Native Hawaiian/Other Pacific Islander)]*	1 (2.1%)	0 (0%)	χ^2^ = 0.00, *p* = 1.000
* [n(%White)]*	21 (43.8%)	44 (95.7%)	χ^2^ = 27.28, *p* < 0.001
* [n(%Other)]*	1 (2.1%)	0 (0%)	χ^2^ = 0.00, *p* = 1.000
* [n(%Multiracial)]*	13 (27.1%)	1 (2.2%)	χ^2^ = 9.62, *p* = 0.002
*Handedness [n(%Right)]†*	35 (72.9%)	39 (84.8%)	χ^2^ = 0.42, *p* = 0.518
*Physical characteristics*			
^ *a*^*Growth Deficiency*	7 (14.6%)	4 (8.7%)	χ^2^ = 0.32, *p* = 0.571
^ *b*^*Microcephaly*	6 (12.5%)	0 (0%)	χ^2^ = 4.23, *p* = 0.040
^ *c*^*Dysmorphic Face*	12 (25%)	2 (4.3%)	χ^2^ = 6.36, *p* = 0.012
*FASD Diagnosis*			
* FAS [n(%FAS)]*	2 (4.2%)	*NA*	
* PFAS [n(%pFAS)]*	11 (22.9%)	*NA*	
* ARND [n(%ARND)]*	35 (72.9%)	*NA*	
* ADHD Diagnosis [n(%ADHD)]*	30 (62.5%)	1 (0.02%)	χ^2^ = 38.68, *p* < 0.001
*Stimulant use [n(%Prescribed stimulant medication)]*	21 (43.8%)	2 (0.04%)	χ^2^ = 19.73, *p* < 0.001

*PAE* Prenatal alcohol exposure group

^†^Handedness information was not available for 7 participants (5 PAE, 2 comparison)

^a^Height or weight ≤ 10%ile

^b^Head circumference ≤ 10%ile

^c^At least two of the following: Palpebral fissure length ≤ 10%ile, thin vermillion border, smooth philtrum (4 or 5 on lipometer scale). The two comparison group participants who had “dysmorphic faces” had scores of 4 on the philtrum and 4 on the vermillion border; neither had any other facial features nor abnormal growth parameters

Notably, as is common in clinical populations, alcohol-related neurobehavioral disorder (ARND) represented the majority of the diagnoses within the PAE group (*n* = 35), followed by partial fetal alcohol syndrome (PFAS; *n* = 11) and fetal alcohol syndrome (FAS; *n* = 2), respectively. There was no significant group difference in within-scanner motion as estimated with Euler values, *t* (92) = 1.44, *p* = 0.153. One participant in the PAE group was previously enrolled in a choline supplementation trial. We did not specifically collect information about other participants’ diets or nutritional supplementation. In addition, a significantly greater number of PAE participants than comparison participants were taking stimulant medications at the time of the study. Participants were not told to stop taking their medication. As expected, participants in the PAE group had significantly lower performance across neurocognitive domains of IQ, episodic memory, and working memory than comparisons (Table 2). We followed the advice of Dennis et al. [54] and did not include IQ as a covariate in our analyses because the lower IQ is directly due to the PAE itself and comparing the groups at values of the covariate that are unrepresentative of the population with FASD would be problematic.

**Table 2:** Neurocognitive test performance by group

	PAE (*n* = 48)	Non-exposed comparison group (*n* = 46)	Statistical Test
*Intelligence Quotient*^*1*^* [M (SD)]*	92.7 (15.1)	115.4 (12.2)	*t*(89) = −8.006, *p* < 0.001
Episodic *Memory*			
*PSMT*^*2*^* [M (SD)]*	95.3 (19.5)	111.5 (16.8)	*t*(91) = −4.303, *p* < 0.001
*Working Memory*			
*Digit Span*^*1*^* [M (SD)]*	8.51 (3.06)	10.9 (2.60)	*t*(90) = −4.009, *p* < 0.001
*LSMT*^*2*^* [M (SD)]*	42.6 (14.5)	49.6 (8.39)	*t*(92) = −2.853, *p* = 0.005

^1^WISC-V

^2^NIH Toolbox

### Group differences in hippocampal thickness and gyrification

**Fig. 2:**
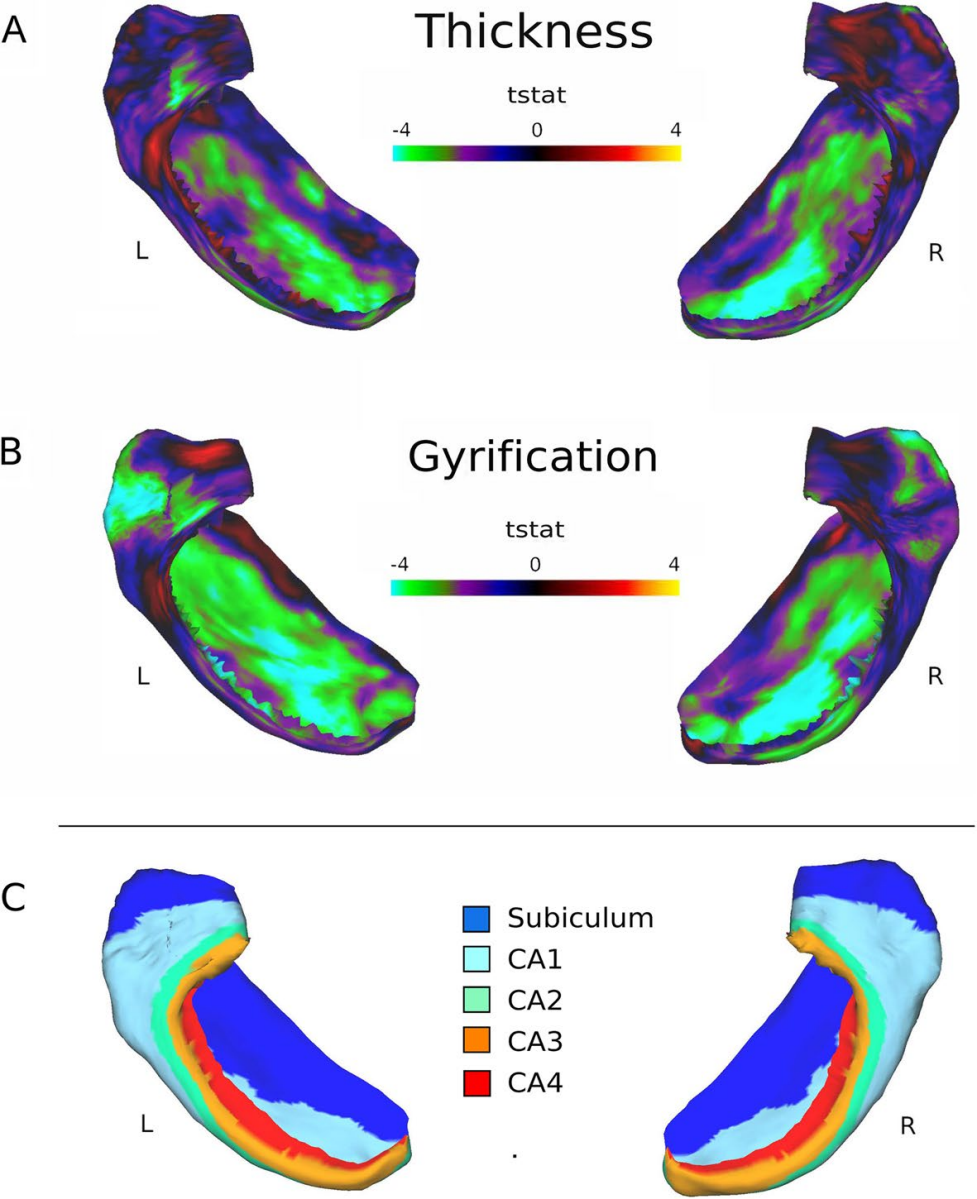
Group differences in hippocampal thickness and gyrification. *Note:* Permutation analysis results showing vertex-wise group differences (PAE vs. comparison) in hippocampal thickness (**A**) and gyrification (**B**). Color intensity shows unthresholded t-statistics (scaled identically for both metrics) at each vertex on the hippocampal surface. In this instance, greens and violets indicate thinner hippocampal tissue and lower gyrification (folding) and reds and yellows indicate thicker tissue and greater gyrification for the PAE group compared to the comparison group. Panel C contains a map of the hippocampal subregions for reference

Vertex-wise permutation analysis revealed significant regional group differences (PAE vs. comparison) in thickness and gyrification across both the left and right hippocampus. Participants with PAE demonstrated thinner hippocampi compared to unexposed comparison participants in portions of the subiculum and CA1 regions while other areas of thickness were similar across groups (Figs. 2 & S1). In addition, participants with PAE showed lower gyrification in widespread portions of CA1 and subiculum in the bilateral hippocampus (Figs. 2 & S2).

### Relationship of hippocampal thickness and gyrification to cognitive performance

**Fig. 3:**
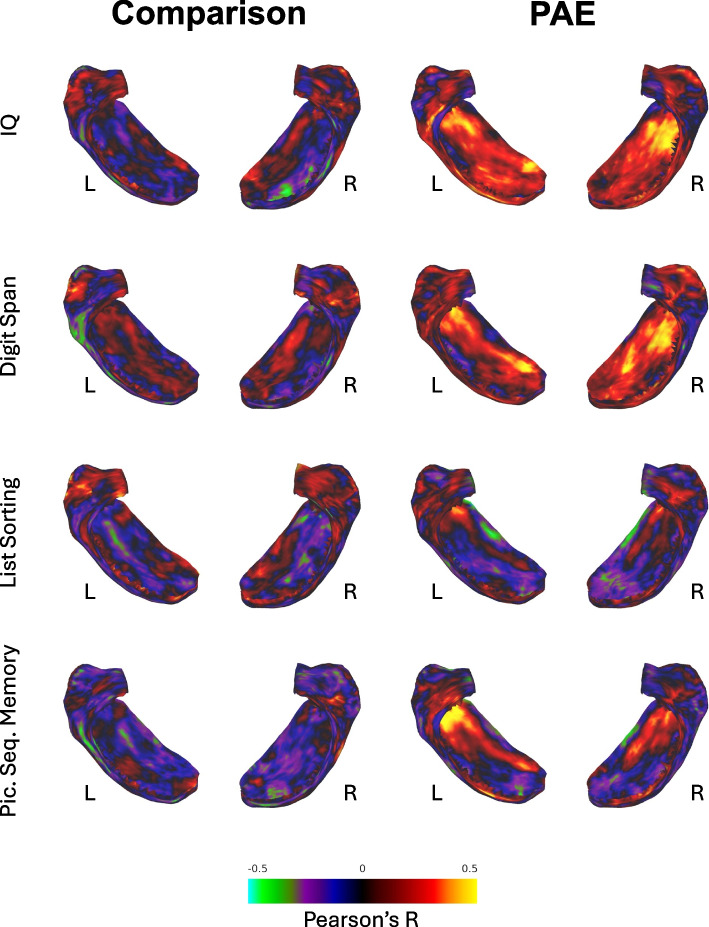
Correlation of hippocampal thickness to neurocognitive performance. *Note:* Permutation analysis results showing vertex-wise correlations between hippocampal thickness and neurocognitive performance. Color intensity shows unthresholded Pearson’s R values at each vertex on the hippocampal surface. Greens and blues would indicate negative correlations between thickness and cognitive functioning (none present) and reds and yellows indicate positive correlations. IQ, Wechsler Full-Scale IQ standard score; Digit Span, Wechsler Digit Span scaled score; Picture Sequence Memory, NIH Toolbox Picture Sequence Memory Test T-score; List Sorting, NIH Toolbox List Sorting Working Memory Test T-score

For participants in the PAE group, significant positive correlations were observed between IQ and bilateral hippocampal thickness in the subiculum (Figs. 3 & S3). Working memory performance (Digit Span) was significantly positively correlated with subiculum thickness in the right hippocampus (Figs. 3 & S4), while episodic memory performance (PSMT) was significantly and positively correlated with left subiculum thickness, most prominently in the anterior aspect (i.e., when visualizing the folded hippocampus) (Figs. 3 & S5). In contrast, for participants in the unexposed comparison group, correlation maps revealed no significant associations between hippocampal thickness and cognitive functioning.

**Fig. 4:**
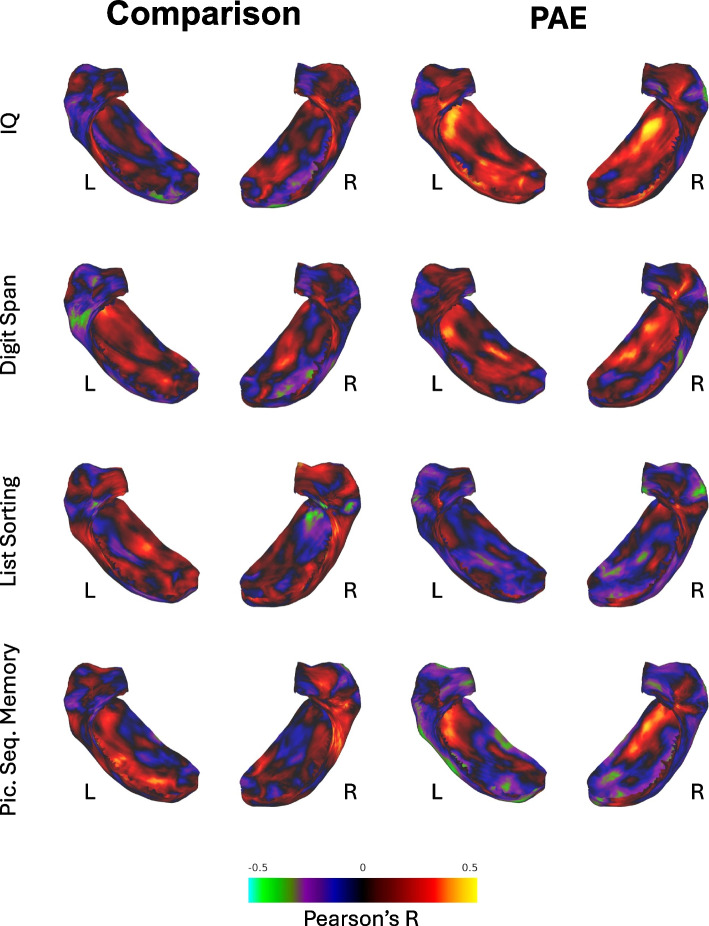
Correlation of hippocampal gyrification to neurocognitive performance. *Note:* Permutation analysis results showing vertex-wise correlations between hippocampal gyrification and neurocognitive performance. Color intensity shows unthresholded Pearson’s R values at each vertex on the hippocampal surface. Greens and blues would indicate negative correlations between gyrification and cognitive functioning (none present) and reds and yellows indicate positive correlations. IQ, Wechsler Full-Scale IQ standard score; Digit Span, Wechsler Digit Span scaled score; Picture Sequence Memory, NIH Toolbox Picture Sequence Memory Test T-score; List Sorting, NIH Toolbox List Sorting Working Memory Test T-score

There were minimal to no statistically significant correlations between hippocampal gyrification and cognitive performance for either participants with PAE or unexposed comparison participants (Fig. 4) with the exception of small regions of positive (non-significant) correlations between gyrification and IQ in anterior aspects of the bilateral subiculum for those with PAE.

## Discussion

This study highlights abnormalities in regional hippocampal thickness and gyrification in a sample of youth with PAE compared to unexposed peers. These data build on previous published work by employing a newly-available MRI tool designed for hippocampal “unfolding” in order to characterize hippocampal morphology at a vertex-wise level. This approach complements and builds upon previous work demonstrating hippocampal anomalies in PAE via measurement of whole hippocampal volume or regional sub-volumes. The vertex-wise approach here allows for a detailed analysis of hippocampal structure as well as the measurement of individual differences in hippocampal folding [38], providing new insights into alcohol’s impacts on an important formative developmental process (folding) that has not previously been analyzed in youth with PAE.

Participants with PAE demonstrated prominently thinner bilateral hippocampi in CA1, which is consistent with previous findings of reduced volume of CA1 in youth with PAE when using norm-adjusted and age-specific volumetric z-scores for hippocampal subfields [42]. The current findings are also consistent with data from animal models, which have repeatedly demonstrated smaller CA1 volume in those exposed to alcohol prenatally [28, 55–57]. In the current study, participants with PAE also demonstrated thinner hippocampi in portions of the subiculum, which aligns with previous work showing reduced volume of this subfield in youth with PAE [42]. The subiculum is an important subfield that functions as a gateway to the entorhinal, perirhinal, and prefrontal cortices as well as to other subcortical regions including the hypothalamus [58]. The subiculum receives direct input from CA1 and may play an important role in organizing informational output from the hippocampus [59]. It is also known to inhibit activity of the hypothalamic–pituitary–adrenal axis [58, 60], suggesting it may play a role in stress regulation and emotional control. There were no group differences in thickness across other hippocampal subfields, which suggests that the teratogenic impact of PAE may have some regional specificity within the hippocampus. The hippocampus, along with other brain regions such as the corpus callosum, cerebellum, and caudate, are thought to be particularly vulnerable to the teratogenic insult of PAE through a number of mechanisms including apoptosis (programmed cell death), altered gene expression, oxidative stress, and abnormalities in trophic and growth factors, among others (for reviews see [61, 62]). More recent work using animal models of PAE has also implicated neuroinflammation as an additional insult to the developing hippocampus, including activation of microglia and increased expression of pro-inflammatory cytokines [63]. Together, a combination of these alcohol-related effects produce damaging effects on hippocampal structure and function that may impact affected individuals throughout the lifespan.

In addition to thinner regional hippocampal tissue in parts of CA1 and the subiculum, participants with PAE also demonstrated lower gyrification (i.e., folding of the hippocampus) in more widespread bilateral portions of CA1 and subiculum regions. This may reflect the teratogenic effect of PAE, which attenuates the normal developmental process of hippocampal thickening and, in turn, impacts the intricate folding of the hippocampus during early development. Evidence from animal models indicates that PAE causes significant neuronal loss differentially across the hippocampal subfields and that the degree of loss is dependent upon the timing of the exposure [56]. In addition, animal models also show that PAE induces neuroinflammation and alters myelination and synaptic plasticity in the developing hippocampus [63]. During gestation, the hippocampus forms initially as flat tissue that progressively folds onto itself [20], including wrapping around the dentate gyrus and “folding” in the anterior–posterior plane. This process of hippocampal folding is similar to the folding patterns seen in the neocortex, which undergoes a protracted developmental process of rapid gyrification during the third trimester [64] that peaks during the toddler years and then begins to decline [65]. This folding/gyrification process results in a significantly greater surface area (and therefore volume) of the cortex than would occur without folding–a process known to be vulnerable to the teratogenic insult of PAE. Indeed, atypical cortical gyrification in this population has been described in several cross-sectional studies [66–68] and one longitudinal study [69]. Future studies will benefit from further exploration of regional hippocampal gyrification anomalies in individuals with PAE—including longitudinal examinations of developmental trajectories of hippocampal folding—particularly during childhood when the hippocampus is undergoing considerable subfield-specific developmental changes [40].

Abnormalities in regional hippocampal morphology may play a role in the well-known learning and memory deficits that occur in children with PAE, including impaired verbal learning, recall, and discrimination [11]. These deficits are likely to be meaningful and lifelong as suggested by studies in which adults with FASD commonly report frequent memory difficulties [31]. In the current study, we observed abnormally thinner and flatter hippocampi in the CA1 region in children with PAE compared to the typically developing group. In both human and animal models, CA1 is implicated in long-term episodic memory [70, 71]. We were not able to detect statistically significant associations between the CA1 abnormalities and cognitive performance in our participants. However, we did find several correlations between regional hippocampal structure and neurocognitive performance in select domains. Specifically, for children with PAE, thicker hippocampal tissue in the bilateral subiculum was associated with better IQ. Also in the PAE group, a thicker right subiculum was associated with better working memory performance, and a thicker left subiculum was associated with better episodic memory performance. These findings are perhaps consistent with a proposed model of subiculum function based on underlying neuroanatomical connections and animal behavioral studies that suggests it serves both a stress regulatory role (via the hypothalamic–pituitary–adrenal axis) and a role in memory and spatial perception [58]. Linking regional structural hippocampal anomalies with neurocognitive functioning in specific domains, as well as identifying relationships between hippocampal structure with functional connectivity in networks subserving neurocognitive performance (e.g., between the hippocampus and neocortex; for example as shown by [72]), will be important aims for future research.

In contrast to the findings for the PAE group, for children in the unexposed comparison group, there were minimal associations between hippocampal thickness and neurocognitive functioning. This may reflect reduced covariation or range restriction given that unexposed individuals are expected to generally perform well on neurocognitive tests and to show typical hippocampal development. Relationships between hippocampal gyrification (i.e., folding) and cognitive performance were not significant for PAE participants or unexposed comparisons. Notably, in a previous study of individuals with PAE ages 8 to 16 years, hippocampal subfield volumes were not found to be associated with episodic memory performance [42], which may reflect the greater regional specificity and detailed measurement of thickness of the hippocampus used in the current study (and the fact that the complex morphology of the hippocampus was not accounted for in the previous study).

Overall, these new findings suggest that individuals with PAE have atypical hippocampal development that is associated with worse neurocognitive functioning. The data suggest that specific regions (especially CA1 and subiculum) may be particularly vulnerable to developmental insults caused by PAE. These insights may provide direction to the design of neurodevelopmental interventions for individuals with PAE such as the incorporation of spatial memory training, working memory assistance, and even stress regulation as it relates to learning. These insights could also potentially guide the development of biological interventions as well. For example, a number of randomized controlled trials of the nutrient choline have been conducted in PAE and follow-up studies are underway [33–36, 73–75]. Because choline is known to play an important role in hippocampal development and function, a careful examination of the hippocampi (including the subfields highlighted here) in those who have received choline supplementation as an intervention may be warranted.

## Limitations and future directions

This study has limitations that are important to acknowledge. The sample size was modest and it is possible that some neurodevelopmental effects attributable to PAE may have been missed and some associations between anatomical differences and neurocognitive abnormalities may not have been detected. Nonetheless, the data did yield evidence of anomalies that have apparent clinical relevance, and the study demonstrated the potential of the novel methodology. Because the study was designed to evaluate a broad range of cognitive functioning—including IQ, working memory, and episodic memory—it did not include an exhaustive evaluation of all possible memory domains. Rather, we were limited to examining associations between hippocampal morphology and a single measure of short-term sequential episodic memory functioning (NIH Toolbox Picture Sequence Memory Test) and two measures of working memory (Wechsler Digit Span and NIH Toolbox List Sorting Working Memory Test). Future studies examining hippocampal morphology would benefit from a more detailed assessment of memory and learning processes including memory recall and recognition following a delay. In addition, evaluating regional hippocampal morphology in both younger children and adults with PAE and FASD including longitudinal investigations may reveal altered developmental trajectories specific to individual subfields. In our sample, there were significant differences in race between PAE and unexposed comparison groups, with more non-White participants in the PAE group, which may limit the generalizability of the findings to some degree. For example, research has linked lower socioeconomic status (SES, which is associated with race and racist oppression) with smaller volumes and atypical growth trajectories of the hippocampus and other subcortical structures, which may relate to exposure to stress during early development (for review see [76, 77]). In addition, some participants in our sample were taking stimulant medications at the time of their cognitive evaluations, which may have a modest positive effect on cognitive testing in domains such as working memory and episodic memory [78]. However, given the size of this subgroup, we were unable to explore this statistically. In this study, we did not have details of the birth parents’ socioeconomic status (SES), race, education, occupation, or income and, therefore, we did not attempt to quantify the participants’ SES. It is also important to acknowledge that participants in the study were exposed prenatally to other substances of abuse in addition to alcohol as is common in the population of affected individuals. We were not able to statistically control for or otherwise analyze the impact of these other substances and, therefore, this must be recognized as a limitation of the study. As such, we acknowledge that the hippocampal anomalies described here are likely the result of many known and unknown factors influencing both pre- and postnatal neurodevelopment including PAE, other exposures, SES, and other related factors. Future research will benefit from continued efforts to increase equitable access to research participation and inclusive recruitment practices.

Regarding the clinical translation of our findings, it is important to note that individual differences in hippocampal folding could not be easily translated for clinical use in most settings, as it would require access to an MRI scanner and expertise in analyzing structural MRI images. However, academic health centers and academic children’s hospitals may have the necessary technology and expertise to collect hippocampal folding metrics (and other neuroimaging metrics) and use them in clinical evaluations. In the future, these neuroimaging metrics might be used as additional measures that are combined with neuropsychological test scores and parent-report measures for some children with PAE or suspected PAE (and potentially other neurological and neurodevelopmental conditions) to aid diagnosis or assess the effectiveness of interventions.

## Conclusions

Using a novel method of characterizing hippocampal structure, we demonstrate altered hippocampal thickness and gyrification in youth with PAE compared to an unexposed comparison group. We also show that abnormal thinning of the hippocampal in youth with PAE is associated with neurocognitive impairment.

## Supplementary Information

Supplementary Material 1.

## Data Availability

The datasets used and/or analyzed during the current study are available from the corresponding author on reasonable request. Additional information can be found at cifasd.org.

## Abbreviations

ARND: Alcohol-related neurodevelopmental disorder
FAS: Fetal alcohol syndrome
FASD: Fetal alcohol spectrum disorders
D-KEFS: Delis-Kaplan Executive Functioning System
PFAS: Partial fetal alcohol syndrome
PAE: Prenatal alcohol exposure
WISC-V: Wechsler Intelligence Scale for Children, 5th Edition
LST: NIH Toolbox List Sorting Working Memory Test
PSMT: NIH Toolbox Picture Sequence Memory Test
