# Supplementary material for: Regional hippocampal thinning and gyrification abnormalities and associated cognition in children with prenatal alcohol exposure
Source: J Neurodev Disord. 2025 Feb 5;17:5. doi: 10.1186/s11689-025-09595-8 (PMC11796126; doi:10.1186/s11689-025-09595-8)

Supplementary Material

Figures S1-S5 highlight the vertices which reached significance (95% CI) after familywise error correction and threshold-free cluster enhancement. Unthresholded *t*-statistic and Pearson *r*-statistic maps are presented in Figures 2-4 of the main text.

S1. Thickness: PAE < Comparison (see Fig 2A)

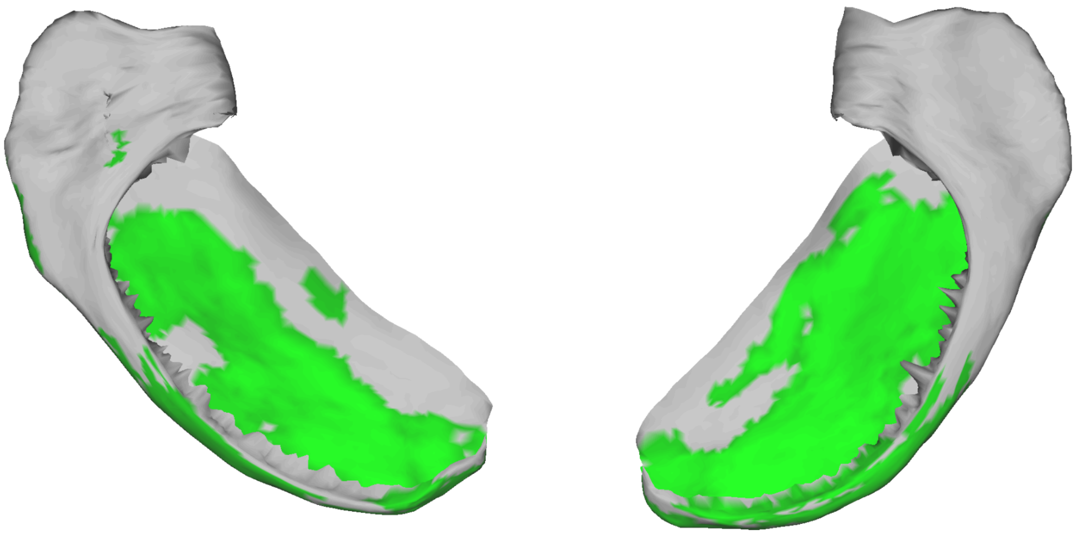

S2. Gyrification: PAE < Comparison (see Fig 2B)

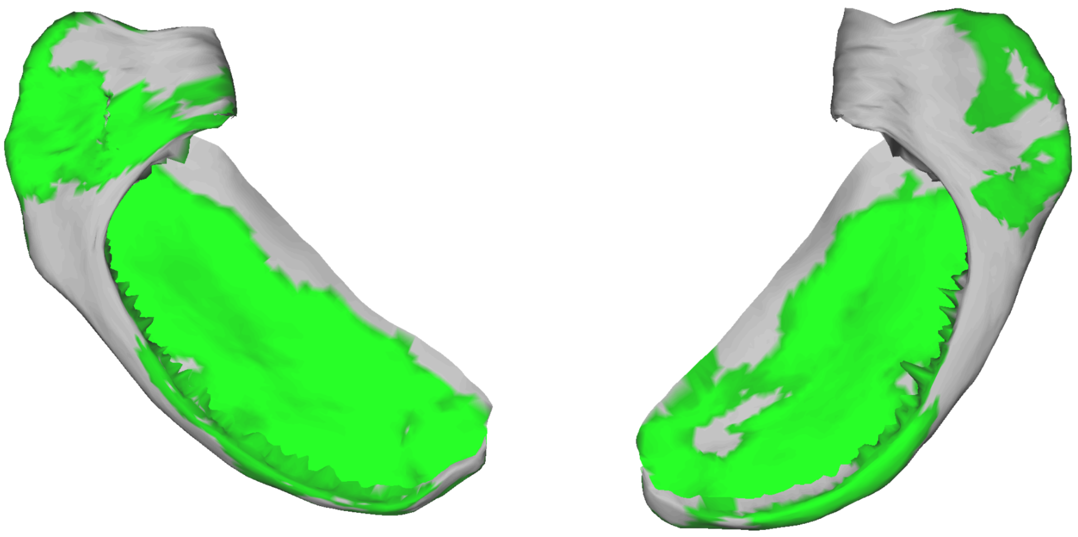

S3. Correlation (+) between thickness and IQ in PAE group (see Fig 3, right column, row 1)

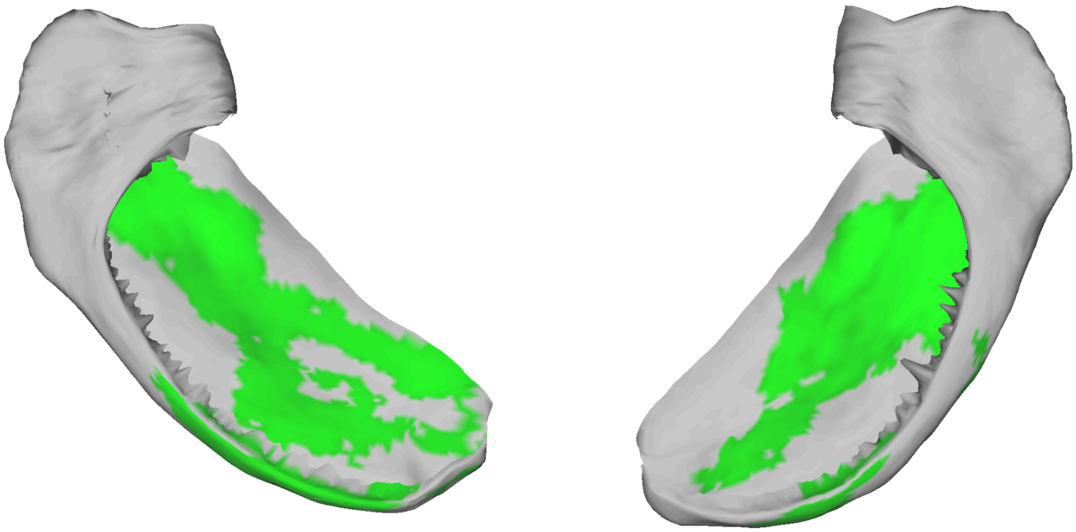

S4. Correlation (+) between thickness and Digit Span score in PAE group (see Fig 3, right column, row 2)

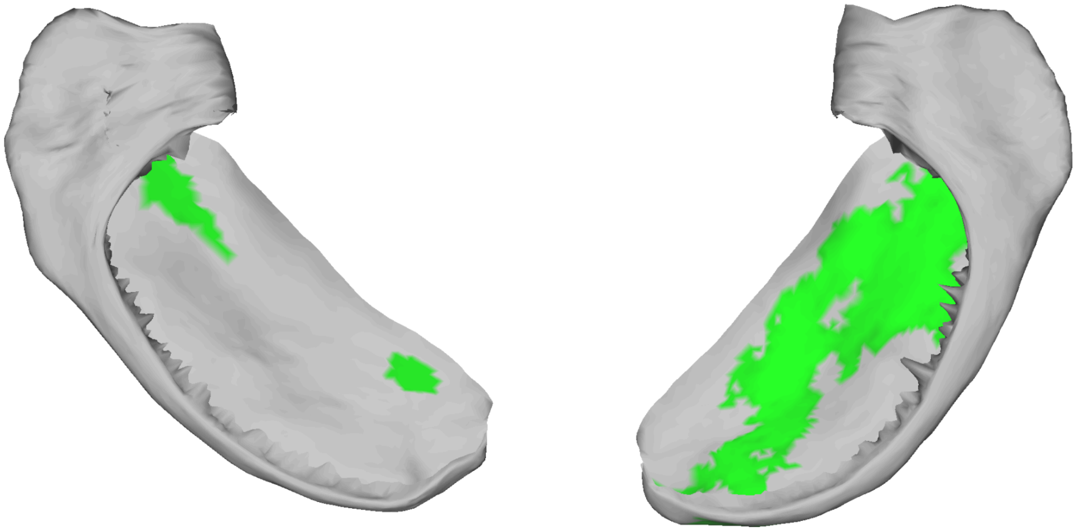

S5. Correlation (+) between thickness and Picture Sequence Memory score in PAE group (see Fig 3, right column, row 4)

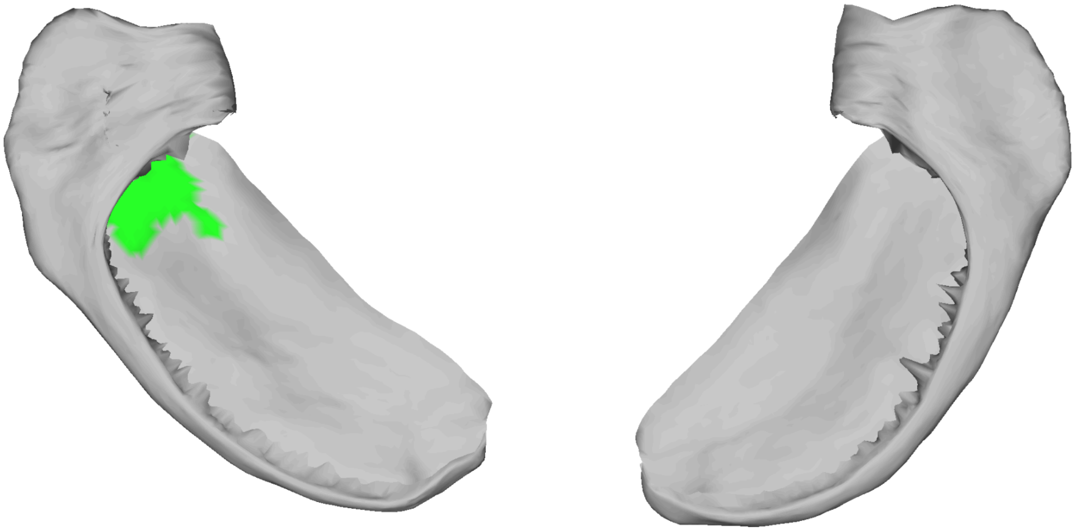

Supplement: Supplementary file 1 — Supplementary Material 1. [file 11689_2025_9595_MOESM1_ESM.pdf]
